# Phylogeny of Zika Virus in Western Hemisphere, 2015

**DOI:** 10.3201/eid2205.160065

**Published:** 2016-05

**Authors:** Robert S. Lanciotti, Amy J. Lambert, Mark Holodniy, Sonia Saavedra, Leticia del Carmen Castillo Signor

**Affiliations:** Centers for Disease Control and Prevention, Fort Collins, Colorado, USA (R.S. Lanciotti, A.J. Lambert);; Department of Veterans Affairs, Palo Alto, California, USA (M. Holodniy);; VA Caribbean Health Care System, San Juan, Puerto Rico, USA (S. Saavedra);; Laboratorio Nacional de Salud Guatemala, Villa Nueva, Guatemala (L. del Carmen Castillo Signor)

**Keywords:** Zika virus, flavivirus, New World, viruses, phylogeny, Puerto Rico, Guatemala, Western Hemisphere

**To the Editor:** Zika virus belongs to the genus *Flavivirus*, family *Flaviviridae,* and is transmitted by *Aedes* spp. mosquitoes. Clinical signs and symptoms of human infection include fever, headache, malaise, maculopapular rash, and conjunctivitis. 

Zika virus was first isolated in 1947 from the blood of a febrile sentinel rhesus monkey during a study of yellow fever in the Zika Forest of Uganda (*1*). During the next 20 years, Zika virus isolates were obtained primarily from East and West Africa during arbovirus surveillance studies in the absence of epidemics. During those 20 years, cases of Zika virus infection were detected sporadically; however, given the clinical similarity of Zika virus and dengue virus infections and the extensive cross-reactivity of Zika virus antibodies with dengue viruses, it is possible that Zika virus was associated with epidemics that were incorrectly attributed to dengue viruses. Beginning in 2007, substantial Zika virus outbreaks were reported first in Yap Island (Federated States of Micronesia), then in French Polynesia, and then in other Pacific Islands (*2*–*4*).

Genetic studies have revealed that Zika virus has evolved into 3 distinct genotypes: West African (Nigerian cluster), East African (MR766 prototype cluster), and Asian. It has been postulated that the virus originated in East Africa and then spread into both West Africa and Asia ≈50–100 years ago (*5*). In early 2015, cases of Zika virus infection were detected in Rio Grande State, northern Brazil, and limited sequence analyses revealed that the virus was most closely related to a 2013 isolate from French Polynesia, within the Asian clade (*6*). 

In December 2015, the Centers for Disease Control and Prevention Arbovirus Diagnostic Laboratory detected Zika virus in serum specimens collected from persons in Guatemala and Puerto Rico. The complete nucleotide sequence of the virus was derived directly from 3 of these serum specimens by using next-generation sequencing on the Ion Torrent (Thermo Fisher Scientific, Waltham, MA, USA) platform. The raw sequence reads were analyzed and assembled by using the CLC bio Genomics Workbench (CLC bio, Waltham, MA, USA) and Lasergene NextGen (DNAStar, Madison, WI, USA). The complete genome sequences were aligned by using ClustalW (http://www.megasoftware.net/) with all available full-length Zika virus sequences from GenBank representing the 3 genotypes. Nearly identical phylogenetic trees were generated by using several methods (minimum-evolution, maximum-likelihood, neighbor-joining), and a neighbor-joining tree was generated and analyzed with 1,000 replicates for bootstrap testing (Figure). GenBank accession numbers for ZIKV sequences presented in this article are KU501215 (Puerto Rico PRVABC59), KU501216 (Guatemala 8375), and KU501217 (Guatemala 103344).

**Figure:**
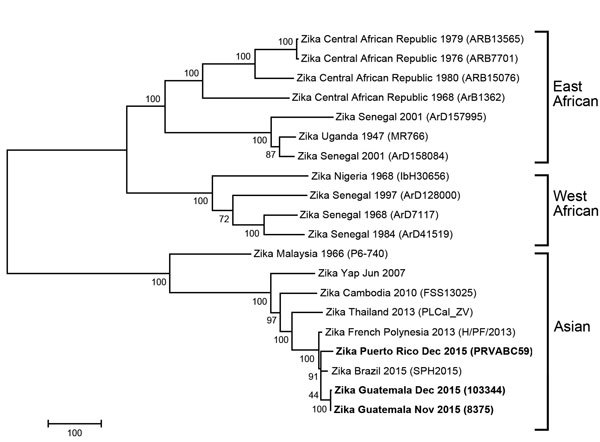
Phylogenetic tree of Zika virus isolates identified from Guatemala and Puerto Rico in December 2015 (indicated in boldface) compared with reference isolates obtained from GenBank. The isolates from Guatemala and Puerto Rico grouped with other Asian genotype viruses. The tree was derived by neighbor-joining methods (bootstrapped 1,000 times) using complete-genome sequences. Location, year identified, and GenBank strain identification for the viruses used in tree construction are shown. Scale bar indicates number of nucleotide substitutions per site. GenBank accession nos.: KU321639 (Brazil 2015 SPH2015), KJ776791 (French Polynesia H/PF/2013), KF383115 (Central African Republic ARB1362), KF383116 (Senegal 1968 ArD7117), KF383117 (Senegal 1997 ArD128000), KF383118 (Senegal 2001 ArD157995), KF383119 (Senegal 2001 ArD158084), KF268948 (CAR 1979 ARB13565), KF268949 (CAR 1980 ARB15076), KF268950 (CAR 1976 ARB7701), EU545988 (Yap 2007), KF993678 (Thailand 2013 PLCal_ZV), JN860885 (Cambodia 2010 FSS13025), HQ234499 (Malaysia 1966 P6-740), HQ234501 (Senegal 1984 ArD41519), HQ234500 (Nigeria 1968 IbH 30656), LC002520 (Uganda 1947 MR766), KU501215 (Puerto Rico PRVABC59), KU501216 (Guatemala 8375), and KU501217 (Guatemala 103344).

In agreement with the initial sequencing of samples from Brazil conducted by Zanluca et al. (*6*), the 3 newly sequenced Zika viruses from Guatemala and Puerto Rico are all within the Asian genotype and most closely related to strains recently isolated from Brazil (2015) and French Polynesia (2013). The tree topology confirms previous findings and indicates that Asian genotype viruses have been gradually evolving and spreading geographically throughout Asia and the Pacific Islands since at least 1966; the tree suggests that the Malaysia 1966 isolate is representative of an ancestral genotype (*7*). The percent nucleotide identity among all the Western Hemisphere Zika viruses is >99%, and as a group, these Western Hemisphere viruses are ≈89% identical (96% aa) to viruses of the East African and West African genotypes. 

As reported by Musso et al. (*8*), the phylogeny and movement of Zika and chikungunya viruses are strikingly similar. Each virus is grouped into 3 genotypes of very similar geographic distribution: East Africa, West Africa, and Asia. For both viruses, it also seems that viruses from East Africa moved into Asia ≈50–100 years ago and evolved into a unique Asian genotype (*9*,*10*). In addition, the similarity with respect to the recent movement of these viruses from Asia into the Pacific Islands and then into the New World (*9*) is noteworthy. It seems that similar ecologic and/or human social factors might be responsible for the movement of chikungunya and ZIKV viruses into the New World at approximately the same time. Further studies might elucidate the exact mechanism of this transcontinental movement, leading to effective prevention strategies.
